# Experiences of family violence and parental unavailability in childhood in relation to parental socioeconomic position and psychological problems: a cohort study of young Swedish women 1990–2013

**DOI:** 10.1186/s12905-021-01292-7

**Published:** 2021-04-09

**Authors:** Jesper Löve, Kirsten Mehlig, Åsa Källström, Gunnel Hensing, Hrafnhildur Gunnarsdottir

**Affiliations:** 1 School of Public Health and Community Medicine, Institute of Medicine, University of Gothenburg, Box 453, 405 30 Gothenburg, Sweden; 2 School of Law, Psychology and Social Work, Örebro University, 701 82 Örebro, Sweden; 3 Department of Health Sciences, University West, 461 86 Trollhättan, Sweden

**Keywords:** Socioeconomic position, Parental unavailability, Parental rejection, Parental lack of time, Family violence, Childhood, Women

## Abstract

**Background:**

Despite the high prevalence and severe consequences for health and wellbeing, epidemiological research of neglected emotional needs during childhood is scarce and little is known about its relation to parental socioeconomic position (SEP). This study investigates the prevalence of family violence and parental unavailability in childhood and its association with parental SEP and parental psychological problems in four strata of young Swedish women examined 1990, 1995, 2000, and 2013.

**Method:**

The sample comprised 976 women (mean age 22, range 20–25) living in Sweden. Secular trends for family violence, parental rejection and unavailability were analyzed using logistic regression as a function of year of examination. The associations with parental SEP and parental psychological problems were assessed using logistic regression with results in terms of odds ratios (OR) and 95% confidence intervals.

**Results:**

Gendered patterns were observed in the associations between parental psychological problems and family violence and parental unavailability. Maternal psychological problems were associated with maternal rejection OR 6.8 (3.5–13.0), maternal lack of time OR 2.4 (1.2–5.0), and paternal rejection OR 1.9 (1.1–3.5). Paternal psychological problems were associated with paternal rejection OR 4.0 (2.1–7.7), paternal lack of time OR 4.9 (2.3–10.6), and experiencing family violence OR 4.9 (2.1–11.6). Low and medium parental SEP were associated with experience of family violence in childhood OR 3.1 (CI 1.1–8.5) and OR 3.4 (1.7–6.9), respectively. No changes between 1990 and 2013 were observed for the prevalence of any of the outcomes.

**Conclusions:**

A stable prevalence of family violence and parental unavailability was reported by young women examined between 1990 and 2013. Lower socioeconomic position was associated with family violence while the association with parental unavailability was non-significant. Gendered patterns were observed in the association between parental psychological problems and family violence, where paternal but not maternal psychological problems were associated with family violence. Further, maternal psychological problems were associated with paternal rejection while paternal psychological problems were not associated with maternal rejection. Gendered patterns of parental unavailability need further studies.

## Background

From a life course perspective, childhood is an important time for the development of cognitive ability, self-confidence, life opportunities, and future health. Therefore, parents should establish a good relationship with their children, including providing for their children’s physical, emotional and educational needs [1–3]. Child maltreatment is any act of commission or omission that causes harm, the potential for harm, or a threat of harm to a child [4]. Acts of commission, which can affect the child directly and/or indirectly, include physical, sexual, or psychological abuse directed towards the child or an intimate partner. In this study, we refer to such acts as family violence. The acts of omission include failure to meet a child’s basic physical, medical/dental, educational, or emotional needs [4] which we refer to as parental unavailability in this study. Parents who do not fulfil their relational role increase the risk for their children to be affected by anxiety, posttraumatic stress, psychosis, self-inflicted injuries, suicide attempts, eating disorders, obesity, alcohol risk use, drug use, and criminality and arrests, with strong evidence for some outcomes and inconsistent evidence for others [4–6]. By threatening the security of attachment, adverse interactions with parents (e.g., neglect of emotional needs) may contribute to the development of internal working models of self and self-in-relation to others (e.g., vulnerability to harm, shame, and self-sacrifice) that influence later cognitive schemas and psychological adjustment, which in turn increase the risk of mental disorders [7]. Particularly, emotional neglect has been found to have severe short- and long-term harmful effects on children’s well-being and development [8], such as mental disorders [9], internalizing symptoms, substance use behaviors [10], and lower health-related quality of life [11, 12]. Although few studies have examined this issue, one study has found that more than 18% of all children internationally suffer from some kind of neglect during their childhood [13], and a recent German study observed that more than 7% suffered from severe emotional neglect [14]. Despite a high prevalence [4, 13, 14] and connection to severe physical and sexual abuse [4, 6], epidemiological research of neglected emotional needs during childhood and potential long-term consequences is lacking [4, 13, 15]. Moreover, studies including emotional neglect often treat it as a by-product as few studies have focused on this outcome directly [13]. In order to promote children’s positive development, health and wellbeing, it is important to early detect families that cannot meet children’s emotional needs.

Children’s basic emotional needs include attention, acceptance and accomplishment. Children need to be seen and heard, accepted for whom they are and feel that they attain some goals of success, and healthy attachment relationships early in life are necessary to provide for these emotional needs [16, 17]. Neglecting the emotional needs of a child differs from neglecting physical needs (including failure to provide adequate nutrition, clothing, supervision, personal hygiene, and medical attention) and educational needs (including permitting chronic absence from school and ignoring special educational needs) [13]. Glaser [18] highlights the multidimensionality of emotional neglect and state that when defined it should be “based on the elements that comprise a child’s psychosocial being” [18, p.703]. He describes following five dimensions: 1) failure to promote the child’s social adaptation; 2) failure to recognize and acknowledge the child’s individuality and psychological boundary; 3) use of developmentally inappropriate or inconsistent interactions with the child; 4) use of negative attributions and misattributions directed at the child and finally; 5) emotional unavailability and unresponsiveness. Recognizing a child’s individual needs demands that parents are physically available during enough time in their child’s life and emotionally available to be able to acknowledge those needs. This is the dimension in focus of this study.

There exists different and potentially complementing causal models for why parents end up neglecting the needs of their children. Whereas psychological researchers have mainly focused on individual characteristics of the parents (e.g., the parental deficit model), sociological researchers have mainly focused on the parents’ social and economic situation (e.g., the environment deficit model) [19]. As a combination of the above-mentioned models, the ecological-transactional model focuses on how familial and individual attributes interact with contextual factors in relation to neglect [19]. Both perspectives have received some support. For example, Yaghoubi-Doust [20] observed an association between neglect and parental substance abuse and Drake and Pandey [21] observed an association between neglect and neighborhood poverty status. Further, Lacey et al. [22] found poverty associated with all kinds of childhood abuse and neglect and Hartras [23] claims that childhood maltreatment overall “mainly are manifestations of poverty, deprivation and gender inequality” [p. 440]. To prevent neglect (e.g., by providing support for parents who struggle in their parenting), more knowledge is needed about the conditions related to different aspects of emotional neglect. Although an association between parental socioeconomic position (SEP) and child maltreatment (i.e., using indicators like injuries and violent and un-intentional deaths) have been observed [24], less is known about the relation to neglect of emotional needs. This knowledge gap includes both the mechanisms and existence of such an association. An empirical study from the UK has challenged the existence of an association between economic and educational disadvantage and emotional neglect: “Claims that families who are poor or are less well educated do not engage in high profile “good” parenting practices are misplaced” [25] [p.138]. However, associations might include more indirect mechanisms than negative parental practices such as higher prevalence of mental adversities in parents. Although some studies have investigated the relationship between living conditions during childhood and emotional neglect, few have investigated the roles of psychological and socioeconomic struggles in the family and to our knowledge no epidemiological studies have been conducted in a Swedish context that focus on these issues.

### Children’s living conditions in Sweden

In 1979, a law was passed in Sweden that prohibits all forms of corporal punishment, including by caretakers and parents, directed at children. In 1990, Swedish legislation ratified the United Nations Convention on the Rights of the Child, in which states accept an obligation to respect, protect and fulfil the rights of children. The Convention includes children’s right to physical, psychological, spiritual, moral and social development (article 6), for which parents have primary responsibility [26]. In 1993, the Swedish government appointed an ombudsman to protect children’s rights and interests. As a result of these measures, in 2010, Sweden was ranked number one among developed countries on Save the Children’s “Children’s Index Rank”; the best country to be a child [27]. Thus, in Sweden, like in many other countries, the official and public awareness of children’s rights has developed significantly during the last 40 years. Less is known about changes in the levels of child adversities in Sweden during these years. Despite this development 8.6% of Swedish men and 13.1% of Swedish women reported in 2011 that they had experienced physical or emotional neglect during their childhood [5]. A higher proportion of young women (9.9%) than young men (4.9%) reported that they had experienced serious concerns or were sad or had worried that they did not have anyone in their lives that could help them, listen to them, comfort them, take their concerns seriously, and protect them against threats when they were a child [5]. Although children in Sweden on average might have a positive living situation, inequalities exist and children with parents of lower socioeconomic position and/or with a migration background often face larger challenges [28, 29]. However, researchers and society have yet to acknowledge these disparities, including the potential relationship between parental socioeconomic position, the psychosocial situation of families, and the risk for neglect of emotional needs. From a public health perspective, increased knowledge in this field might contribute to the design of future social policies and interventions that will decrease the number of children exposed to these kinds of adversities.

This study investigates the development over time of family violence and parental unavailability in childhood, among young adult women in Sweden. In addition, the aim was to investigate family violence and parental unavailability in relation to parental socioeconomic position and parental psychological problems.

## Method

### Study design

This study was based on data from the population-based project “Women and Alcohol in Gothenburg” (WAG) [30]. The WAG project had a two-stage design with an initial screening survey (13 items) addressing alcohol-related problems sent to all women registered in the central districts of Gothenburg [30]. This procedure was conducted in four waves between 1989 and 2013 and included women born in 1965, 1970 (first wave in 1989), 1975 (second wave in 1995), 1980 (third wave in 2000), and 1993 (fourth wave in 2013). All women with five or more alcohol-related problems, 25% of the women with one to four problems, and 15% of the women without any problems were invited to participate in a face-to-face interview. In the 2013 screening, all women participating in the screening were also invited to the face-to-face interview. The subsequent interview, conducted by clinically experienced health-care personnel, consisted of comprehensive questions on work, family, childhood, and health-related issues [31].

### Study population

The sample analyzed in the current study included all women born in 1965, 1970, 1975, 1980, and 1993 who had participated in a baseline face-to-face interview (N = 978). The mean age at the time of the interview was 22.4 years (range 20.0—25.9 years).

## Measures

### Family violence

Experience of family violence during childhood was measured with the question “Have you ever, before the age of 18 years, experienced that someone in your family was beaten or maltreated?” The response alternatives were “yes” or “no”.

### Parental unavailability

Parental unavailability was measured with the questions about parental lack of time and parental rejection, using the following questions:

“Did you perceive that your father had enough time for you when you were 0–12 years old? When you were 13–18 years old?” and “Did you perceive that your mother had enough time for you when you were 0–12 years old? When you were 13–18 years old?” Response alternatives were “yes” or “no”. Parental lack of time was defined as participants responding that their mother or father did not have enough time for them at ages 0–12 and/or 13–18. “Can you tick in the number that best describes your father’s approach to you during your childhood?” and “Can you tick in the number that best describes your mother’s approach to you during your childhood?”. The respondents then chose a number between 1 (Accepting) and 5 (Rejecting). The variable was dichotomized into accepting (score 1–2) and rejecting (score 3–5).

### Parental socioeconomic position

Socioeconomic position was based on an open-ended question about parental occupation and the answers were categorized into low (i.e., non-skilled and skilled blue-collar, no university education), medium (white-collar < 3 years university education), and high (white-collar ≥ 3 years university education) social position. Occupational codes from Statistics Sweden [32] were used and the categorization was based on the occupation of the parent who had the highest level of occupation. The coding and categorization are in line with the Erikson-Goldthorpe scheme [33], which considers both educational and occupational aspects of social position.

### Parental psychological problems

Parental psychological problems were assessed as a combination of two questions: “Did any of your parents have a mental disorder or mental problems (not alcohol) that seriously affected the situation in the family and that they sought treatment for?” (“yes” or “no”) and “Looking back at your childhood, how often did the following persons consume alcoholic brewages?” (five-point Likert scale from “never” to “had problems with alcohol”). Paternal psychological problems were defined as responding “yes” to the first question and/or choosing the alternative “had problems with alcohol” for father. The equivalent procedure was used for maternal psychological problems.

### Statistical analysis

Because three of the four study waves (1990–2000) included an intended oversampling of women with alcohol problems, weighting procedures were applied to obtain representative results for the source population. That is, each participant was given a weight that was inversely proportional to the probability of having been sampled from the source. Descriptive statistics include weighted proportions for family violence, each item of parental unavailability and their 95% confidence interval (CI). In 2013, all women were included irrespective of their screening result and all observations received equal weight, which was set to one in the pooled analysis of all cohorts. Secular trends for family violence and each item of parental unavailability were analyzed using weighted logistic regression as a function of the examination year. *P*-values for a linear trend across years are given for the unadjusted model and after adjustment for age at interview. A pooled data set was used to limit potential bias due to low power in analyses of associations. Based on the pooled data set, the prevalence of family violence and each item of parental unavailability were presented in relation to parental SEP and parental psychological problems. These associations were further examined using weighted logistic regression adjusting for age and year of examination, and the results were given in terms of odds ratios (OR) for family violence and each item of parental unavailability with 95% CI. Statistical analyses were performed with SAS version 9.4 (SAS Institute, Cary, NC). The figure was prepared using MATLAB version R2016b (The MathWorks Inc.).

## Results

### Prevalence of family violence and the parental unavailability from 1990 to 2013

We found no statistically significant changes in the prevalence of family violence, parental rejection or lack of time for the data from 1990 through 2013 (Table 1). In the pooled sample, the highest prevalence was found in paternal lack of time (40.5%) followed by maternal lack of time (27.2%), paternal rejection (18.4%), experience of family violence (10.4%), and maternal rejection (8%).

**Table 1:** Prevalence of family violence and parental unavailability (rejection and lack of time) 1990–2013

Wave (year)^a^	1990	1995	2000	2013		Pooled sample (1990–2013)
N	93	418	294	171		976
Mean age (range)	24.8 (23.8–25.9)	23.0 (20.0–25.9)	21.2 (20.2–25.9)	21.6 (20.8–22.6)		22.4 (20.0–25.9)

	% (95% CI)	% (95% CI)	% (95% CI)	% (95% CI)	*p*^b^	% (95% CI)
Maternal rejection	10.1 (4.5–15.8)	8.2 (5.0–11.4)	6.6 (3.4–9.8)	5.8 (2.3–9.4)	0.09	8.0 (5.8–10.2)
Paternal rejection	11.5 (5.7–18.4)	19.8 (14.2–25.3)	20.3 (14.9–25.7)	15.0 (9.5–20.4)	0.6	18.4 (14.8–22.1)
Maternal lack of time	–	–	29.5 (22.9–36.2)	20.5 (14.4–26.6)	0.07	27.2 (22.0–32.4)
Paternal lack of time	–	–	41.3 (34.3–48.3)	38.2 (30.9–45.6)	0.5	40.5 (35.0–46.1)
Family violence	–	–	10.9 (6.7–15.1)	8.8 (4.5–13.1)	0.4	10.4 (7.0–13.7)

% = weighted prevalence, *95% CI* 95% confidence intervals

^a^Range (in years) for each wave of data collection: 1989–1991, 1994–1998, 2000–2002, 2013–2015

^b^*p* value for linear trend with adjustment for age

**Fig. 1:**
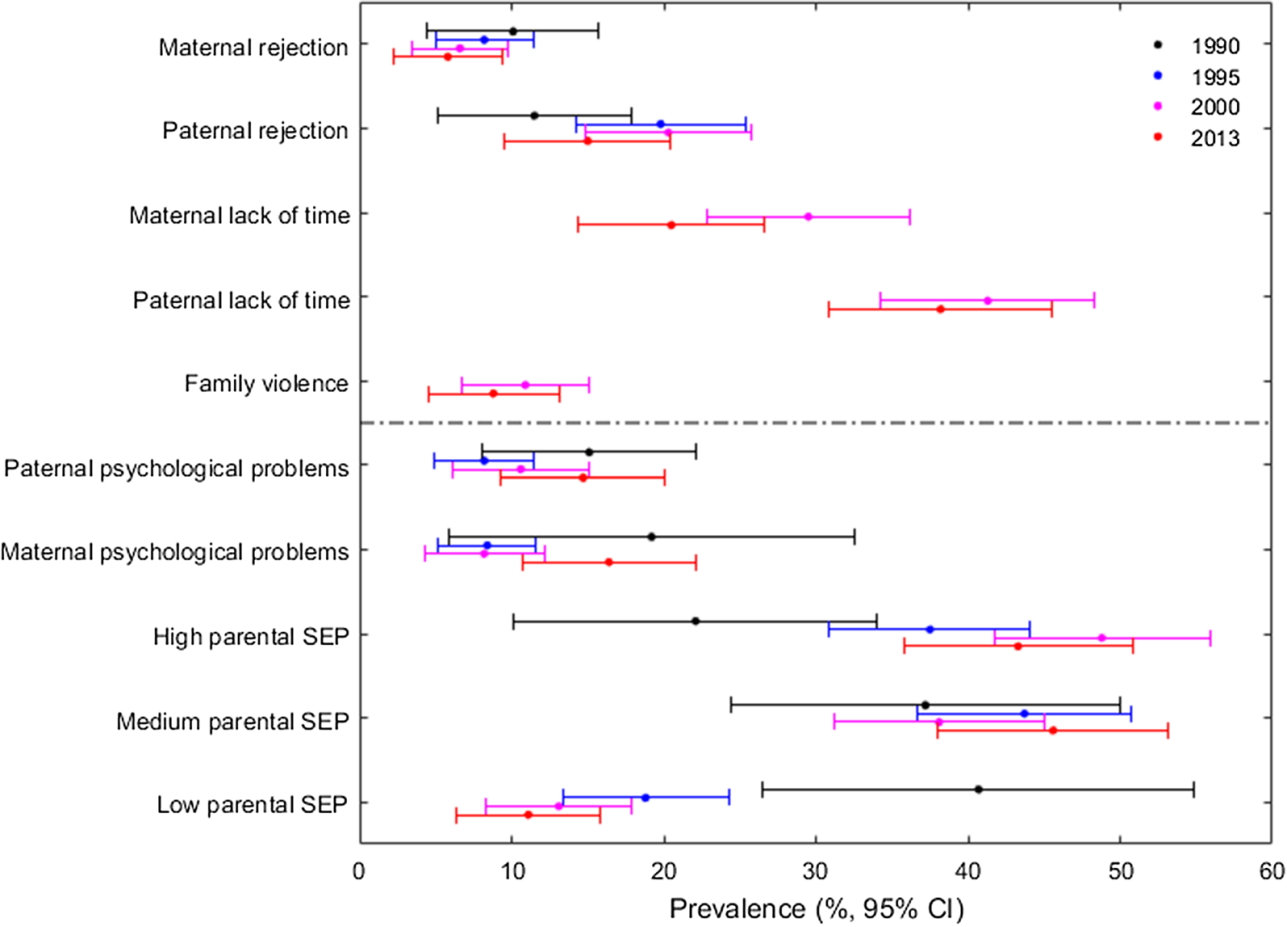
Weighted prevalence of family violence, parental unavailability, psychological problems, and socioeconomic position 1990–2013

The lower panel of Fig. 1 gives the prevalence of childhood living conditions: the prevalence of women with low SEP was higher in 1990 than in 1995, 2000 and 2013 and vice versa, the prevalence of women with high parental SEP was lower in 1990 than in 1995, 2000 and 2013. Apart from that, the women’s childhood living conditions were similar in all the cohorts.

### Parental socioeconomic position in relation to family violence, and parental unavailability

Experiencing family violence was higher for women with low (14.1%) and medium (15.2%) parental SEP than for women with high parental SEP (5.3%). After adjusting for cohort and age at interview, we found that the odds to experience family violence in childhood were three times higher in women with low compared to high parental SEP (last column in Table 2). Women of parents with high parental SEP more frequently reported paternal lack of time compared to women of parents with medium or low parental SEP, however, this difference was not found statistically significant in the regression analysis. No differences of parental SEP were observed for experience with parental rejection (Table 2).

**Table 2:** Parental unavailability and family violence in relation to parental socioeconomic position (SEP) and psychological problems

	Maternal rejection (n = 966)		Paternal rejection (n = 946)		Maternal lack of time^a^ (n = 464)		Paternal lack of time^a^ (n = 462)		Family violence^a^ (n = 463)	
	%	OR (95% CI)^b^	%	OR (95% CI)^b^	%	OR (95% CI)^b^	%	OR (95% CI)^b^	%	OR (95% CI)^b^
*Parental SEP*										
High	7.6	Ref	17.9	Ref	25.0	Ref	46.8	Ref	5.3	Ref
Medium	7.0	0.96 (0.45–2.05)	18.0	1.02 (0.60–1.72)	29.0	1.25 (0.70–2.24)	34.6	0.61 (0.37–1.00)	15.2	3.39*** (1.67–6.90)
Low	10.4	1.43 (0.6–3.38)	22.0	1.33 (0.68–2.61)	30.6	1.31 (0.57–3.00)	36.5	0.66 (0.32–1.35)	14.1	3.07* (1.11–8.52)
*Maternal psychological problems*										
No	5.5	Ref	17.4	Ref	25.1	Ref	39.0	Ref	10.6	Ref
Yes	28.2	6.76*** (3.51–13.0)	29.0	1.94* (1.08–3.48)	43.2	2.43* (1.18–5.00)	45.9	1.37 (0.69–2.73)	13.5	1.38 (0.60–3.17)
*Paternal psychological problems*										
No	7.3	Ref	15.4	Ref	25.6	Ref	34.9	Ref	8.0	Ref
Yes	12.3	1.78 (0.82–3.88)	42.1	4.00*** (2.09–7.66)	30.3	1.38 (0.67–2.86)	71.2	4.89*** (2.25–10.6)	28.8	4.92*** (2.09–11.6)

% = weighted prevalence, *OR* odds ratios, *95% CI* 95% confidence intervals

^a^Based on data from 2000 to 2013

^b^Weighted logistic regression models including age and year of examination

*p* values *p < .05; **p < .01; ***p < .001

### Parental psychological problems in relation to family violence and parental unavailability

Compared to women who had mothers with no psychological problems, women who had mothers with psychological problems reported to greater extent that they experienced maternal unavailability, both in terms of rejection and lack of time (Table 2). In addition, among women who had a mother with psychological problems the odds to experience maternal rejection were seven times higher and the odds to experience maternal lack of time were two times higher compared to women who had a mother without maternal psychological problems. Maternal psychological problems were not associated with experiencing family violence or with fathers’ lack of time, but women with a mother who had psychological problems had two times higher odds to experience a rejecting father.

Women who had a father with psychological problems reported to greater extent that they experienced paternal unavailability, in terms of both rejection and lack of time, and family violence compared to women who had a father without psychological problems. After adjusting for cohort and age at interview, we found that women who reported paternal psychological problems during childhood had five times higher odds to experience family violence or perceive their father as being unavailable in terms of both rejection and lack of time. No associations were seen between paternal psychological problems and maternal unavailability.

## Discussion

This study sets out to investigate 1) the development over time of family violence and parental unavailability in childhood 2) family violence and parental unavailability in relation to parental socioeconomic position and parental psychological problem, among young adult women in Sweden. The results do not support any trends in the prevalence of either family violence or parental unavailability for the study period (1990 to 2013). In the pooled analyses, the prevalence of the five included variables ranged from 8% for maternal rejection, to above 40% for paternal lack of time. The results support an association between lower parental socioeconomic position and family violence. While, no association was observed between socioeconomic position and parental rejection, a weak association between parental socioeconomic position and paternal lack of time was found. The study also added support for an association between parental psychological problems and experiences of parental unavailability, although with some gendered patterns.

### Experiences of family violence in relation to parental SEP and psychological problems

About 10% of the women in the pooled data sample reported experiencing family violence during their childhood. Based on the formulation of the question in the study, we cannot say whether the participants were the target of the violence or whether they witnessed physical violence of another family member. The role of experiencing family violence for children’s health and wellbeing, both in long and short term, has received increased attention [4, 34, 35]. In this study, family violence was found related to parental SEP: women of parents with low or medium SEP had three times higher odds to report family violence than women of parents with high SEP. The association between low SEP and experiences of family violence can be considered in congruence with Hartras [23] claims about childhood maltreatment being a manifestation of poverty and disparity whereas the association between medium SEP and family violence indicate more complex mechanisms. Font et al. [36] concluded it not being “just” about poverty, based on their results showing that neglect was related to substantially worse outcomes when compared to no neglect among youths despite levels and duration of poverty. Since the late 1970s, researchers have developed typologies that distinguish differences between perpetrators and characteristics of the violence. These typologies suggest that while some family violence is embedded in a pattern of attempting to dominate one’s partner by severe and frequent acts of violence (primarily male batterers with alcohol abuse and/or mental problems), the most common type of family violence is less severe violence assumed to occur in specific emotional situations [37, 38]. Assuming that stress such as financial struggles could lead to strong emotions, this study found that family violence was related to lower parental SEP, suggesting that the family violence reported by the women was primarily of this less severe form, although more common. However, the results indicating that the experience of family violence was also related to paternal rather than maternal psychological problems may suggest that the mechanism reflects violence associated with a male batterer. Although this situation may be less common, it is worth noting that children living in such situations may be used as tools in the violence as perpetrators often use the attachment between the mother and child to control them [39]. One may hypothesize that the first type of violence discussed here relates primarily to the environment deficit model and the latter type of violence relates primarily to the parental deficit model described in the introduction, both views supported by our results.

### Parental unavailability in relation to parental SEP and psychological problems

In this study, parental unavailability was captured by parental rejection and lack of time. More women reported paternal rejection than maternal rejection at all time points after 1990, and in 2013 the prevalence of experiencing a rejecting father was three times higher than for a rejecting mother. Rejection from a father or a mother has previously been observed to increase the risk of childhood sexual abuse [40] and psychological maladjustment in adulthood [41]. The results also showed that a rejecting father was associated with the prevalence of psychological problems in either the mother or the father, whereas a rejecting mother was only associated with psychological problems in the mother. These results resemble Bancroft et al.’s findings, which suggest that fathers perpetrating emotional neglect in the form of family violence generally show comparatively little interest in and involvement with their children, acting often as an authoritarian, neglecting and verbally abusing their children [42]. However, these fathers may also reflect Cater et al.’s finding [34] that young women report more parental verbal aggression than young men. The authors hypothesize that gendered differences in emotional expression and interpretation may be responsible for young women being more able to recognize subtle nonverbal and verbal behavior as rejection. However, it is unclear whether child neglect should be framed as a child’s unmet needs (child focus) or as omissions in parental behavior (parent focus) [43, 44]. Researchers have argued for a child-based definition of neglect, focusing on whether or not the child’s individual needs are met [45]. Our results indicate that this is important, as it may be particularly difficult for parents to understand and acknowledge their child’s needs in specific situations, especially if parents struggle with time pressure or psychological problems.

In this study, the unavailability indicator most frequently reported was parental lack of time. For women born 1980 and 1993, about 40% reported that their father did not have enough time for them during their childhood and 27% reported that their mother did not have enough time for them. Although parental time with children varies between countries, the gendered pattern where fathers spend less time than mothers with their children persists [46]. Time pressure, the subjective perception of not having enough time for everyday life duties, has been proposed to be a major threat for health and wellbeing in modern societies [47]. Parents experience more time pressure than individuals without children [48]; single parents in particular [49]. In a study conducted in 2011, 20% of parents in Sweden reported that they consistently experienced time pressure [50]. The experience of time pressure was more frequently reported among parents with long working hours, young children, and financial difficulties [50]. In the present study, paternal unavailability was more frequently reported by women who had parents with high SEP compared to women who had parents with medium or low SEP. Although not statistically significant, an opposite direction of the association was indicated in relation to maternal lack of time, which seemed to be associated with low parental SEP. This paradox might capture the intersection of gender and SEP with the potential situation where high SEP fathers and low SEP mothers, who often work within the health care sector involving evening and weekend work and sometimes working more than one job, work a lot. Such parental time pressure has been associated with children’s mental health problems, particularly among teenage girls [51] and therefore these working conditions (i.e., the availability of high SEP fathers and low SEP mothers for their children) should be considered. Clearly, time pressure should be considered when designing and creating the favorable conditions for raising children. Nevertheless, it should be noted that lack of time is not an objective measure of exact time spent with children. Rather, the subjective feeling may be related to the felt need in specific situations and/or periods and probably even to social contexts (e.g., how much time parents usually spend with their children in a specific context). Of course, subjective feelings might also apply to gender.

Although previous studies have identified gendered patterns related to parental time with children [52], it is plausible that due to differences in expectations the correlation between actual time spent with children and the perception of parental unavailability may look different in relation to mothers and fathers. In the current study, parental unavailability, both in terms of paternal and maternal rejection and lack of time, was strongly associated to parental psychological problems, including alcohol-related problems. While the knowledge of parental psychological problems and substance abuse being strongly related to various kinds of child neglect have been established in previous research [20], the knowledge about the relations to parental unavailability in form not having enough time is scarce. Thus, the results of this study are important contributions to this area.

### Methodological considerations

The main strength of the study is the multiple waves of data collection over a long period. However, by using the already existing data, validated instrument for capturing the multidimensionality of emotional neglect could not be applied, which can be considered a weakness of the study since it limits the possibility for comparisons. Nevertheless, the study extend the scarce number of epidemiological studies focusing on emotional neglect [4, 13] by investigating parental unavailability in terms of rejection and lack of time, which we consider correspond to the emotional unavailability and unresponsiveness dimension of emotional neglect according to Glaser’s [18] proposed definition. Similarly, no objective measure of parental psychological problems was available. On the other hand, since the measures used were based on the young women’s assessments we can assume that the parents had such pronounced problems that the women were very well aware of these as children. Another limitation is the formulation of the question meant to capture the experience of family violence. That is, the question does not reveal whether the participants were directly abused themselves or whether they witnessed physical violence directed at other family members. In addition, it is reasonable to assume that the respondents primarily answered this question considering violence between the parents, as the total number corresponds well to other studies’ findings of exposure to physical forms of intimate partner violence–e.g., Cater et al., (11.1%) and Annerbäck et al., (12.5%). Child physical abuse tends to be reported at higher rates–e.g., Annerbäck et al., (16.3%) [34, 53]. Based on a previously identified knowledge gap regarding alcohol related issues among women the focus in WAG is on women’s health and living conditions with special focus on alcohol related issues. This can be considered both a strength and a limitation in the case of the present study. A strength since women do report childhood abuse largely than men and thus women’s conditions need special attention. A limitation since the knowledge about neglected emotional needs among men also is scarce, which should be a focus in future research. Further, the participants in the current study had relatively high socioeconomic position, which should not pertain an important threat towards the observed associations. However, the potential underrepresentation of participants with low parental SEP may affect the observed prevalence rates and therefore generalizations of lower SEP should be done with some caution.

## Conclusion

A stable prevalence of family violence and parental unavailability was reported by young women examined between 1990 and 2013. The association with parental socioeconomic position showed an inconsistent pattern. Although no associations were observed in relation to parental unavailability, an association was observed in relation to the experience of family violence with a higher prevalence among participants who had parents with lower socioeconomic position. We also found an association between parental psychological problems and family violence, parental unavailability in terms of both rejection and lack of time, but interestingly these associations were gendered. Paternal but not maternal psychological problems were associated with family violence and maternal psychological problems were associated with paternal rejection while paternal psychological problems were not associated with maternal rejection. Gendered patterns of parental unavailability need further studies.

## Data Availability

The datasets used and analyzed during the current study are available from the corresponding author on reasonable request.

## Abbreviations

SEP: Socioeconomic position
WAG: The project Women and Alcohol in Gothenburg
OR: Odds ratios
CI: Confidence intervals
